# Improved Deep Learning Based Method for Molecular Similarity Searching Using Stack of Deep Belief Networks

**DOI:** 10.3390/molecules26010128

**Published:** 2020-12-29

**Authors:** Maged Nasser, Naomie Salim, Hentabli Hamza, Faisal Saeed, Idris Rabiu

**Affiliations:** 1 School of Computing, Universiti Teknologi Malaysia, Johor Bahru 81310, Malaysia; hentabli_hamza@yahoo.fr (H.H.); idrisrabiu43@gmail.com (I.R.); 2 College of Computer Science and Engineering, Taibah University, Medina 344, Saudi Arabia

**Keywords:** virtual screening (VS), similarity searching, deep learning, deep belief networks (DBN), feature selection

## Abstract

Virtual screening (VS) is a computational practice applied in drug discovery research. VS is popularly applied in a computer-based search for new lead molecules based on molecular similarity searching. In chemical databases similarity searching is used to identify molecules that have similarities to a user-defined reference structure and is evaluated by quantitative measures of intermolecular structural similarity. Among existing approaches, 2D fingerprints are widely used. The similarity of a reference structure and a database structure is measured by the computation of association coefficients. In most classical similarity approaches, it is assumed that the molecular features in both biological and non-biologically-related activity carry the same weight. However, based on the chemical structure, it has been found that some distinguishable features are more important than others. Hence, this difference should be taken consideration by placing more weight on each important fragment. The main aim of this research is to enhance the performance of similarity searching by using multiple descriptors. In this paper, a deep learning method known as deep belief networks (DBN) has been used to reweight the molecule features. Several descriptors have been used for the MDL Drug Data Report (MDDR) dataset each of which represents different important features. The proposed method has been implemented with each descriptor individually to select the important features based on a new weight, with a lower error rate, and merging together all new features from all descriptors to produce a new descriptor for similarity searching. Based on the extensive experiments conducted, the results show that the proposed method outperformed several existing benchmark similarity methods, including Bayesian inference networks (BIN), the Tanimoto similarity method (TAN), adapted similarity measure of text processing (ASMTP) and the quantum-based similarity method (SQB). The results of this proposed multi-descriptor-based on Stack of deep belief networks method (SDBN) demonstrated a higher accuracy compared to existing methods on structurally heterogeneous datasets.

## 1. Introduction

In recent years, chemoinformatics has been an active multidisciplinary research area that is beneficial to chemistry and drug discovery, with the use of various tools and technologies. The use of virtual screening (VS) in chemoinformatics is considered as pertinent to scrutinize records of molecules and identify those structures that are most anticipated to be able to be attached to a drug target. The two main classes of VS are ligand-based and target-based VS [1,2]. Recently, some combinations of both structure-based and ligand-based methods have been introduced [3,4]. In the chemical databases, all ligands are ranked accordingly to their maximum score, and the one with the best score is then subjected to further investigation. The VS is conducted based on structural similarity, searching between known and potentially active ligands, and focusing on the molecular similarity principle, which specifies that molecules with similar structure may have similar activity. Among the most frequently employed procedures for ligand-based VS, similarity searching is commonly used. In this approach, a chemical database is explored to discover the molecules with the closest similarity to a user-defined reference structure [5]. All forms of similarity measures come with three fundamental constituents: (a) the representation, which portrays the structures to be taken into account; (b) the weighting scheme, which designates significance-allocating weights to various sections of the structural representation; and (c) the similarity coefficient, which puts a figure on the level of similarity between two fittingly-weighted representations [6].

The process of measuring the similarity between any two objects involves comparing their features. Molecular features range from physicochemical properties to structural features and are stored in different ways, which are commonly called molecular descriptors. A molecular descriptor is the ultimate outcome of a logical and arithmetical process, which converts data encrypted within a symbolic depiction of a molecule into functional numbers. A molecular descriptor may also be the outcome of a regulated experiment [7]. The performance of 2D fingerprint descriptors, which are frequently employed for accelerated screening during substructure and similarity searches, may involve the use of a fragment dictionary or hashed methods applied to the 2D structural drawings of molecules. This fingerprinting process converts a chemical structure into binary form (a string of “0”s and “1”s), which denotes a kind of chemical shorthand that detects the presence or absence of a certain structural feature in the chemical molecule.

Data fusion is a technique involving the combination of multiple data sources which are translated into a single source, in which the result for the fused source is expected to be more informative compared to the results of the individual input sources [8,9]. The concept of combining multiple information sources has been successfully applied [6] and recent studies have found that, in terms of similarity, more potential actives among top ranking molecules can be identified using fusion of several similarity coefficients than can be obtained by using individual coefficients [10]. In the method proposed in [11], an average set of new rankings is produced by all possible combinations of any number of coefficients for each compound. It was found that, based on the new ranking, in comparison to the best individual coefficient, the best performing coefficient combinations (2 to 8) returned more actives among the top 400 compounds. In general, it is expected that the individual coefficient that excels on its own will be more likely to excel in combinations. Most high performing combinations involved the Russell/Rao, Simple Matching, Stiles, Jaccard/Tanimoto, Ochiai/Cosine, Baroni-Urbani/Buser, and Kulczynski (2) coefficients [11]. There are numerous fusion techniques in the area of information retrieval that can be adapted for chemical information retrieval. Fusion normally involves two basic components: the types of objects to fuse and the fusion technique. In text retrieval, combinations of document representations, queries, and retrieval techniques have been fused using various linear and non-linear methods. In chemoinformatics, molecular representations, query molecules, docking scores, and similarity coefficients have mostly been combined using linear combination techniques [12]. In many fusion experiments, either in text retrieval or chemical compound retrieval, the use of a fused source has shown better results than a single source. In achieving the best retrieval performance through data fusion, two requirements must be taken into consideration: the accuracy of each individual source and the independence of sources relative to one another.

Various techniques have been introduced to reduce dataset dimensionality. One of the best-known techniques developed to deal with data dimensionality is principal component analysis (PCA). This mathematical procedure is used to ensure that the reduction of large dataset variables into smaller sets does not affect the most useful information. The number of (possibly) correlated variables is transformed into a (smaller) number of uncorrelated variables, which are called principal components. PCA is designed to preserve the greatest variability of data and ensure that the data are later translated into new variables that are linear functions of the original dataset. These new variables have a consecutively maximized variance and are uncorrelated with each other [13]. The use of PCA aims to discover relationships between observations, by extracting crucial data information, detecting, and removing outliers, and reducing data dimensionality, in terms of the relevancy of information. All of the aims of PCA are attained by discovering the PCA space, in which the maximum direction of the variance prior to the provided data is represented [14]. The PCA space is comprised of orthogonal principal components (PCs), i.e., axes or vectors. The calculation of the PCs involved in solving the covariance matrix.

Recently, deep learning (DL) techniques have been successfully used in several fields. The learning of parameters in deeper architectures can be a challenging optimization task, similar to that found in a neural network with multiple hidden layers. Hinton et al. [15] suggest that learning in deeper architectures can be conducted in an unsupervised and greedy, layered pattern. The initial input is sensory data, as learning information in the initial layers. The initial layers then train according to the inputs, while their outputs (denoting the initial levels of learned representation) are conveyed as learning information to the secondary layers. Iterations are performed until the desired numbers are acquired, at which point the deep networks are wholly trained. Representations learned in the last layers can be utilized for various tasks. If tasks are of the categorization type, other supervised layers are placed on top of the previous layers while their parameters are learned, both randomly and through the use of supervised data, while the remaining network is set, and the entirety is fine-tuned. The application of DL techniques based on deep artificial neural networks has been a game-changer in the fields of computer vision [15,16,17,18,19,20], speech recognition [21,22,23,24,25,26] and natural language processing [27,28,29,30,31,32]. It is believed that deep learning has brought machine learning closer to achieving its nature goal, which is artificial intelligence [33]. The use of deep learning is known to be beneficial when applying a general-purpose procedure, where features can be learned automatically. The procedure is implemented by involving the deep neural networks (DNNs) of a multilayer stack of simple neural networks with non-linear input-output mappings [22,23,27,34]. Particularly, researchers have investigated the use of one of best deep learning techniques, known as deep belief networks (DBN) as a new way to reweight molecular features and thus enhance the performance of molecular similarity searching, DBN techniques have been implemented successfully for feature selection in different research areas and produced superior results compared to those of previously-used techniques in the same areas [35,36,37].

The DBN’s deep-learning procedure is made up of two phases: layer-wise feature abstraction and reconstruction weight fine-tuning [15]. In the first stage, a family of restricted Boltzmann machines (RBMs) is utilized by the DBN [38] to calculate the layer of reconstruction weights. Later, in the second stage, backpropagation is performed by the DBN to fine-tune the weights that were gathered during the first stage [15], [39]. First, the DBN has been trained on all the molecules in the MDDR datasets to calculate the molecular feature’s weight. Only a few hundred features are then selected, based on their new weight and lowest error rate. In previous studies, the MDDR datasets have been represented by several 2D fingerprints, which include, atom type extended connectivity (ECFP) fingerprints, atom type extended connectivity fingerprint counts (ECFC), functional class daylight path-based fingerprint counts (FPFC), functional class extended connectivity fingerprint counts (FCFC), atom type connectivity fingerprint counts (EPFC), functional class daylight path-based fingerprints (FPFP), *A*Log*P* types extended connectivity fingerprint counts (LCFC), *A*Log*P* types daylight path-based fingerprint counts (LPFC), functional class extended connectivity fingerprints (FCFP), *A*Log*P* types daylight path-based fingerprints (LPLP), and *A*Log*P* extended connectivity fingerprint (LCFP) [40,41,42]. Each of these fingerprints has different important features and different molecular representations. In this study, we implemented a stack of DBNs with each of these molecular fingerprints and only the important features selected from each fingerprint were combined to form a new descriptor, which is used to obtain improved performance of molecular similarity searching for chemical databases. In summary, major contributions of this paper are as follows:

- An improved deep learning method for molecular similarity searching that utilizes feature selection.
- An introduced Stack of DBN method for features reweighting is proposed by emphasizing more weights to the important features.
- The proposed method showed promising results in terms of overall performances in comparison to the benchmark methods.

## 2. Related Works

The core principle of feature selection is to choose a subset of all variables, so that a large number of features with little discriminative and predictive information is eliminated [43,44]. The molecular fingerprints consist of many features; however, not all of them are important. Hence, removing some features can enhance the similarity measure recall [45]. These redundant and irrelevant features would potentially interfere with data mining methods, such as clustering, and make it hard to translate [46]. There are two known approaches for reducing dimensionality, which are feature transformation and feature selection. In feature transformation, a linear or non-linear function is added to the original features to eliminate dimensions; thus, a subset of the original features is selected. In contrast, feature selection is used particularly to preserve the significance of the original features.

Most of the current similarity measures consider all molecular features to be of equal value with the same importance, and all these features are used in calculating similarity measures, which is considered as a drawback. According to Vogt et al. [45], the recall of similarity measure can be enhanced by feature selection, thus allowing more weights to be added on important fragments whilst removing the unimportant ones. Various studies on weighting functions have been carried out by Abdo et al. [47] and a new fragment weighting scheme for the Bayesian inference network in ligand-based virtual screening has been introduced. Ahmed et al. [48] focused on developing a fragment reweighting technique by applying reweighting factors and relevance feedback to improve the retrieval recall of a Bayesian inference network. In comparison to the conventional similarity approaches used, the Bayesian inference network model has enhanced performance and has been widely used in virtual screening as an alternative similarity searching method [49,50,51,52,53].

Various text retrieval studies have shown that, even if the effectiveness of the provided algorithms is similar, the use of different models of retrieval or ranking algorithms will result in low overlap between identified relevant and non-relevant documents [54]. Thus, Turtle and Croft [55] developed an inference network-based retrieval model whereby different document representations and versions of a query are combined in a consistent probabilistic framework. Various researchers have used a range of different methods to combine multiple retrieval runs, which results in better performance than a single run [56]. Moreover, the use of a progressive combination of different Boolean query formulations could potentially lead to a progressive improvements in retrieval effectiveness [57]. The combined effect of using multiple representations was discussed by [58]. Specifically, the INQUERY system was used by Rutgers University [55] and a modified version of the SMART system was used by Virginia Tech. Results from both of these studies showed that the combination methods often led to a better and improved results compared to any individual retrieved set. Another study, conducted on various types of datasets by Ginn et al. [59] used data fusion methods to combine search results, based on multiple similarity measures. They concluded that the application of data fusion offers a simple, but efficient solution to integrate individual similarity measures. Croft et al. [60] investigated a retrieval model that can incorporate multiple representations in accordance to information needs, and found that the combination of different versions of a query gave better performance than the individual versions.

PCA has been used in chemo-informatics in several previous studies. Cao et al. [61] conducted a study on the use of PCA for image acceleration reconstruction in fluorescence molecular tomography (FMT) to overcome the obstacles presented by dimensionality, due to the massive computational load and memory requirement in the inverse problem. Reducing the dimensionality enables faster reconstruction of an image. The result was highly positive, in that the proposed method was able to assist in accelerating image reconstruction in FMT almost without degrading the image quality. A paper by Yoo and Shahlaei [62] discusses different aspects and applications of PCA in quantitative structure activity relationship (QSAR) and suggests that the main purpose of PCA in a typical QSAR is to study the information in the data with regard to interrelationships between descriptors and molecules and decide whether the PCA can integrate related information represented by different structural descriptors into the few first PCs without focusing on a particular data range. Thus, no loss of important information will occur during analysis of the original matrix of descriptors.

A study of DBN techniques has been successfully conducted to perform feature abstraction and reconstruction of images [35,63,64,65]. The results shown an enhancement of the sample classification accuracy on a multi-level feature selection in selecting the least number of the most discriminative genes [66]. The application of DBN has also been conducted on feature selection for remote sensing scene classification [35]. Zhuyun and Weihua [67] proposed using feature fusion based on DBN with multisensory feature fusion for bearing fault diagnosis. The purpose of multisensory information fusion is to produce more reliable and accurate information representations compared with those of single sensor data, because multisensory signals always contain redundant and complementary information which is useful for diagnosis.

Most similarity techniques are based on the assumption that molecular fragments without links to any biological activity are similar in weight to the crucial fragments. Generally, chemists refer to elements in structural diagrams, such as functional groups and place importance on certain fragments over others. Researchers in this domain scrutinize all fragments in the chemical structure of compounds and assign additional weights to the more important fragments. Thus, a match involving two molecules with highly weighted features would be of more significance to the overall similarity than a match involving molecules with low weighted features [68,69]. In addition, using these important features in feature selection improves the performance of the similarity searching. Feature selection is known to be an efficient method for the removal of irrelevant content in data loads, as it delivers an uncomplicated learning model while ensuring reduced time for training and performance of classification. The intricacy of interactions among features, together with the considerable size of the search space, render the selection of beneficial and applicable features difficult. Generally, interactions can result in two different outcomes: (a) the interaction of an irrelevant feature with other features may lead to its relevancy for classifying or learning, and (b) the interaction of a relevant feature with other features may render it dispensable [70]. For this reason, the authors of the present research propose a new method to reweight the molecular features based on stacked deep belief networks with multi-descriptors that determine the important features for each descriptor and combine all these features to introduce a new descriptor for enhancing the performance of the molecular similarity search.

## 3. Materials and Methods

This section presents the methods used in the research, which involve the use of the Stack of deep belief networks method (SDBN) model for molecular similarity searching. We first describe the concept of the DBN method for molecular feature reweighting and then present the proposed SDBN method for molecular similarity searching.

### 3.1. General Structure of the DBN

**Figure 1:**
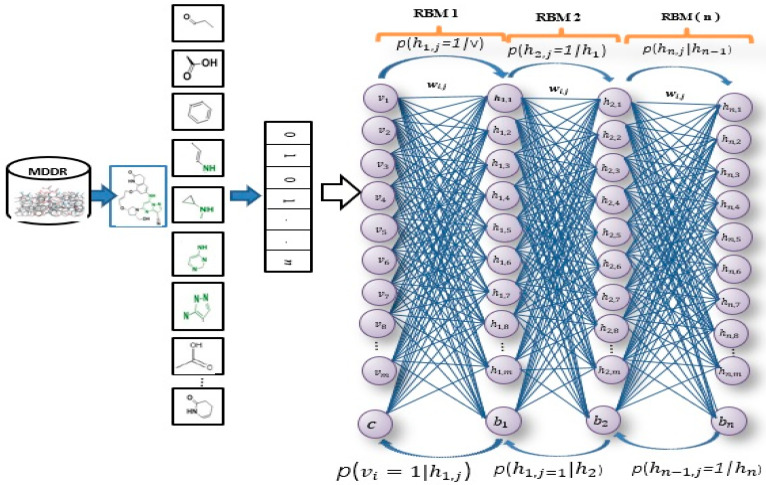
Deep belief networks (DBN) Architecture.

Figure 1 illustrates the general framework of the proposed method using the DBN model. Based on this model, there are two dominant stages, which are layer-wise feature abstraction and reconstruction weight fine-tuning. In the first stage the calculation of the reconstruction weights is done layer-wise, using a family of restricted Boltzmann machines (RBMs). In the second stage, backpropagation is performed to fine-tune the results, based on the weights obtained from the first stage. The reconstruction weight in DBN can be obtained from the layer-wise weights, based on the input features. A reconstruction error can be calculated for each input feature provided, and all layer-wise weights are given. The differences in reconstruction errors are commonly produced based on the variation of features. Normally, features with lower reconstruction error are more re-constructible; thus, they are more prone to retain their intrinsic characteristics. In protein-ligand binding, the key is to identify the feature’s intrinsic characteristics. A selection of the more re-constructible features is proposed as the discriminative features and is later used as an input to a new feature-selection method for ligand-based virtual screening. An iterative procedure of feature learning in the DBN model is performed to remove the feature’s outliers, in order to obtain a reliable reconstruction weight. Large reconstruction errors, which are also known as feature outliers, can be identified by analyzing the distribution of the reconstruction errors. The final reconstruction weight matrix is expected to be more reliable for feature reconstruction, as the feature outliers have been eliminated during the iterative feature learning procedure. The weight matrix attained from training on bioactive compounds is then used to achieve feature selection for further virtual screening. The feature selection is based on a certain threshold after sorting the error values.

As shown in Figure 1, this phase includes a stack of RBMs. The output is obtained from the ${\left(i-1\right)}^{th}$ RBM in the hidden layer, which serves as the input to the visible layer of $i^{th}$ RBM [71]. The output of the lower-level RBM is treated as the input of the higher-level RBM, resulting in an individual training of the RBM, starting from the $i^{st}$ RBM within the stack. Once the first batch of RBMs in the stack finishes the training, the features of the input or an abstract output representation are generated in the hidden layer. These features later serve as the 2nd batch of RBMs in the stack and perform the next training. The procedure continues until the last RBM in the stack finishes its training. RBM is also well-known as an undirected graphical model with two layers [72,73,74], in which the first layer, referred to as the visible units, is comprised of the observed variables, while the second layer, described as hidden units, comprises the latent variable. As it carries the name of Boltzmann Machine [75], RBM is also known as a specific type of log-linear Markov random field (MRF) [76,77]. The differences between the RBM and the general Boltzmann machine is that in an RBM, only inter-layer connections between the visible and hidden units are included. Hence, intra-layer connections between visible-visible, hidden-hidden units are excluded. The architecture stack of the restricted Boltzmann machines is illustrated in Figure 1.

Generally, RBM has the structure of an energy based model that can be used for the visible and hidden layers <v; h> possessing the weights matrix W, that is linked to the connection between v and h [78]. An RBM’s weights and biases dictate how much energy there is in a joint configuration of the visible and hidden units having model parameters θ={W,b} and $v_{i},h_{j}\in \left\{0,1\right\}$, where W represents the symmetric weight parameters that have the following V×H dimensions and b represents the bias parameters. It is interesting to note that the RBM itself is a dependent among the variables. Because an RBM does not have any intra-layer connections, each pair of units in every layer is conditionally independent from another layer. Hence, one can factorize the conditional distributions over hidden and visible units as follows:

(1) $$p\left(v|h\right)=\overset{n}{\underset{i=1}{\prod }}p(v_{i}|h)$$

(2) $$p\left(h|v\right)=\overset{K}{\underset{j=1}{\prod }}p(h_{j}|v)$$

The probability for every unit within the hidden layer $h_{j}$ = 1, where $h_{j}\in \left\{0,1\right\}$ is

(3) $$p\left(h_{j}=1|v\right)=\sigma \left(b_{j}\;+\;\underset{i}{\sum }v_{i},w_{ij}\right)$$

and σ refers to the logistic function with the following definition:

(4) $$\sigma \left(x\right)={\left(1+e^{-x}\right)}^{-1}$$

Similarly, we can calculate the conditional probability of $v_{j}$ = 1 as

(5) $$p\left(v_{i}=1|h\right)=\sigma \left(a_{i}\;+\;\underset{j}{\sum }h_{j},w_{ij}\right)$$

We can obtain the network’s learning rules in the log-likelihood-based training data using alternating Gibbs sampling [16,78]. In Gibbs sampling, every iteration involves an update of every hidden unit in parallel, using Equation (3). All of the units in the visible layer are then updated in parallel using Equation (5) [78]. The derivative of the log probability of a training vector in terms of a weight can be calculated by applying the following formula:

(6) $$-\frac{Ә\log p\left(v\right)}{Ә\;w_{ij}}=\;<v_{i}h_{j}{>}_{data}-\;<v_{i}h_{j}{>}_{model}$$

The angle brackets denote the expectations given within the distribution, specified by the succeeding subscript. This creates a very simple learning rule that can be used to conduct the stochastic steepest ascent for the training data’s log probability:

(7) $$\Delta w_{ij}=\;\varepsilon \left(<v_{i}h_{j}{>}_{data}-\;<v_{i}h_{j}{>}_{model}\right)$$

where parameter ε represents the learning rate. Similarly, the learning rule for the bias parameters can be presented as

(8) $$\Delta a_{i}=\;\varepsilon \left(<v_{i}{>}_{data}-\;<v_{i}{>}_{model}\right)$$

(9) $$\Delta b_{j}=\;\varepsilon \left(<h_{j}{>}_{data}-\;<h_{j}{>}_{model}\right)$$

**Figure 2:**
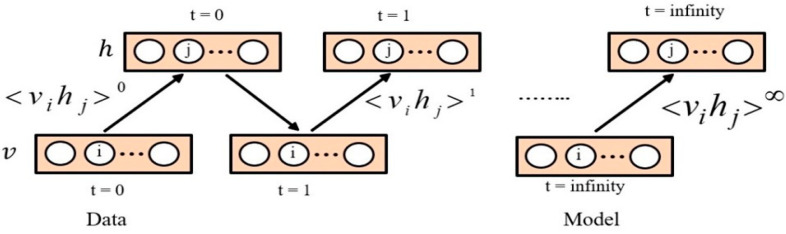
Gibbs Sampling to update all associated weights.

Since no direct connections exist between the hidden units in an RBM, these hidden units can be considered as independent of the visible units [79,80]. Using the above sections as the basis, it is possible to obtain the gradient of log probability of training data using Equation (6). There is a need to calculate $<v_{i}h_{j}{>}_{data}$ and $<v_{i}h_{j}{>}_{model}$ in order to compute the gradient and adjust parameters based on Equation (7). Based on the usage in the majority of the RBMs literature, the calculation of $<v_{i}h_{j}{>}_{data}$ is referred to as the positive phase, while the $<v_{i}h_{j}{>}_{model}$ calculation is referred to as the negative phase. These phases correspond to the positive and negative gradients, respectively. Since no interconnections between hidden units exist, they are considered independent. Thus, we can calculate $<v_{i}h_{j}{>}_{data}$ by taking the visible units 𝑣 (their values have been established by training data) into consideration and giving the value 1 to every hidden unit that has a probability value of $p\left(h_{j}=1|v\right)\;$ with respect to Equation (3). The main issue is found within the negative phase. In practice, the distinction between various DBN learning methods (e.g., persistent contrastive divergence or contrastive divergence) lies in the sampling during their negative phase [80]. To calculate the $<v_{i}h_{j}{>}_{model}$, the Gibbs sampling method was utilized. This method begins by using random values in visible units. The steps of the Gibbs sampling method need to go on for a long time. Each Gibbs sampling step results in an update of all hidden units, based on Equation (3). All visible units are then updated based on Equation (5). The Gibbs sampling is shown in Figure 2, where $<v_{i}h_{j}{>}_{0}$, denotes the expectations for the data distribution and $<v_{i}h_{j}{>}_{k}$ denotes the expectations under the model distribution and ε is the learning rate. Furthermore, the visible or hidden unit activations are considered to be conditionally independent, given hidden or visible units, respectively [78].

### 3.2. The Proposed SDBN Model

This section presents the proposed model, which utilizes the stacked DBN based multi-descriptors method for molecular similarity searching. Eight 2D fingerprints were generated by SciTegic Pipeline Pilot and PaDEL descriptor software [81]. These were 120-bit ALOGP, 1024-bit CDK (CDKFP), 1024-bit ECFC4, 1024-bit ECFP4, 1024-bit path fingerprints (EPFP4), 1024-bit graph only (GOFP) and 881-bit Pubchem fingerprints (PCFP).

The key element of the method is the representation used to translate a chemical structure into mathematical variables. Some descriptors and molecular representations are complementary to other descriptors, and thus could yield better results when used in combination. This means that different descriptors may produce different results for molecular similarity searching. It incorporates several molecular representations in merging and combining the features from multi-descriptors that can improve the performance of the similarity search. In this study, we trained five of these descriptors (ECFC4, ECFP4, EPFP4, Graph, and CDK) and all the combined probability stages have been undertaken as follows:

(1) All stages combine two descriptors (i.e., (ECFC4, ECFP4), (ECFC4, EPFP4), (ECFC4, Grapgh),....)
(2) All stages combine three descriptors ((ECFC4, ECFP4, ECFC4), (ECFC4, ECFP4, Graph), ….)
(3) All stages combine four descriptors ((ECFC4, ECFP4, EPFP4, Graph), (ECFC4, ECFP4, EPFP4,CDK), )
(4) Then, combine five descriptors (ECFC4, ECFP4, EPFP4, Graph, CDK).

**Figure 3:**
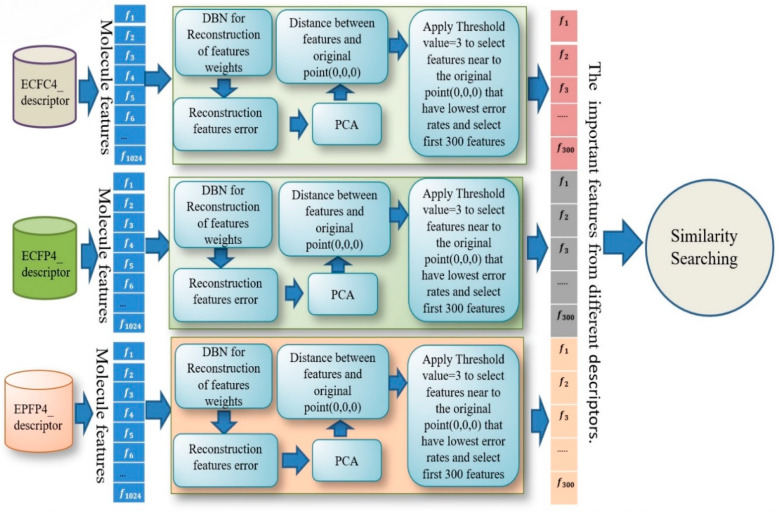
Combine multi descriptors based on SDBN.

After training all these stages, we found that the best results were obtained with the combination of three descriptors: ECFC4, ECFP4, EPFP4. The results of this combination were then used as a new descriptor for the similarity search. The design for combining multi descriptors with DBN for reconstruction of feature weights is shown in detail in Figure 3.

### 3.3. Reconstruction of Features’ Weights

The training of DBN was conducted in both stages (pre-training and fine tune). In this study, DBN was trained with different architectures and different numbers of RBMs. The DBN trained with two RBMs, three RBMs, Four RBMs, and five RBMs successively and with different learning rates (0.01, 0.05, 0.06), and different epochs (20, 30, 50, 70, 100). The weights were randomly initialized between 0 and 1. The configuration that obtained the best results was that using five RBMs (2000, 1800, 1300, 800, 300), 70 epochs and learning rate =0.05. The size of the input layer in the first RBM was 1024; it was similar to the size of the dataset vector with reference to all the molecular features, while the size of the output layer in the first RBM was 2000. After completing the training of the first RBM, the output from the first RBM became the input layer to the second RBM and the size of the output layer was 1800. The third RBM input layer was the output from the second RBM, with size 1800, and the size of the output layer was 1300. Similarly, the output of this RBM was used as the input to the fourth RBM where the output layer size was 800. Finally, the last RBM input layer size was 800 and the output layer was 300. During the training of the RBMs, we used epoch = 70, Gibbs steps = 50, batch size = 128, and learning rate = 0.05. After training in the first stage, backpropagation was used to fine-tune the weights obtained from the first stage “pre-training stage”. The output from this training was pretrained as the new vector with the new weight. For testing, we calculated the reconstruction features’ weight by comparing them with the original features’ weight. Once all the RBMs had been trained and the weights saved for all RBMs, the DBN pre-training was complete. DBN then performed a backpropagation to fine-tune the weights. A reconstruction error was calculated for each input.

**Figure 4:**
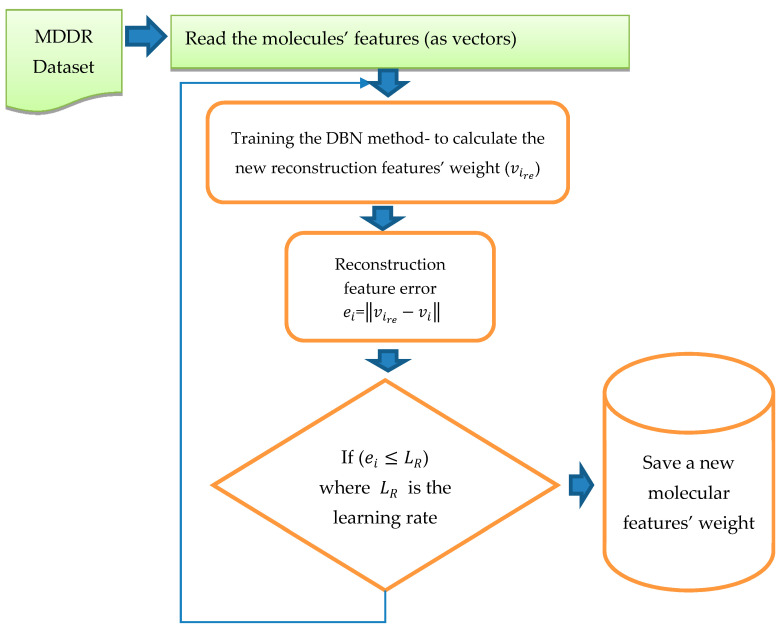
Reconstruction of features weight steps.

Figure 4 shows the process and steps used to obtain the reconstruction features’ weight. The reconstruction error of feature $v_{i}$ was calculated by using $e_{i}$=$\left\|v_{i_{re}}-v_{i}\right\|$, where $v_{i_{re}}$ is the reconstruction feature corresponding to $v_{i\;}.\;$ This new error rate was compared with the error rate calculated from previous training by using $\|e_{i}-e_{i-1}\|\leq e$, where e is the error rate value given to run the code, the inference forward will be used again if $\|e_{i}-e_{i-1}\|>e$. The training was considered to be complete when there was no more change in the error rate and all the weights were fixed; thus it was considered that the network had been learned.

**Figure 5:**
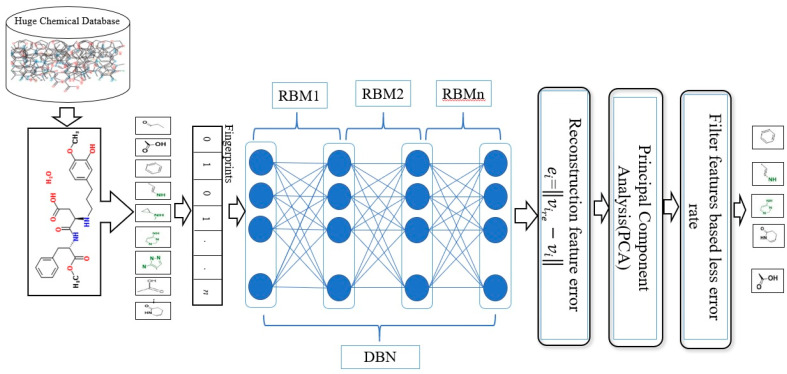
Dimensionality reduction and feature filtering processes.

The next step after training of the DBN is complete, and all new weights for all molecules have been stored, is to apply PCA. PCA has been used to decrease the molecules dimensionality (features) and filter features according to the percentage of reconstruction feature error, as shown in Figure 5. Section 3.4 explains PCA in detail and how it is used in this proposed method.

### 3.4. Principal Component Analysis (PCA)

PCA remains among the fastest algorithmic methods used in the implementation of non-linear dimensional reductions, to decrease the dimensionality of each feature vector while gaining more efficient features [82]. The aim of the PCA technique is to find a lower dimensional space or PCA space that will later be used to transform the data $X=\left\{x_{1},x_{2},x_{3},\ldots ,x_{N}\right\}\;$ from a higher dimensional space, $R^{M},\;$ to a lower dimensional space, $R^{K}$, where N represents the total number of samples or observations and $x_{i}$ represents $i^{th}$ sample, pattern, or observation. All samples have the same dimension $\left(x_{i}\in R^{M}\right)$. This means each sample is assigned by M  variables, i.e., each sample is assigned as a point in M-dimensional space. The direction of the PCA space represents the direction of the maximum variance of the provided data. The PCA space is comprised of K  principal components (PCs). The first PC (($PC_{1}$ or $v_{1}$) ∈$R^{M*1}$) shows the direction of the maximum variance of the data, the second PC has the second largest variance, and so on [83].

In this proposed method, three different types of data sets were trained for the dimension reductions which are MDDR-DS1, MDDR-DS2, and MDDR-DS3. Each data set contained 102516 molecules (samples), and each molecule was represented by 1024 features (variables). We used X to represent the training data set as a training matrix, N is the number of samples or molecules and M is the number of dimensions for each molecule (sample).

(10) $$X=\left[\begin{array}{ccc} x_{1,1} & \cdots & x_{1,M}\\ \vdots & \ddots & \vdots \\ x_{N,1} & \cdots & x_{N.M} \end{array}\right]$$

Each vector in the training matrix X represents one molecule with M features, so that each molecule has 1024 features.

(11) $$V_{i}=\left[\;\begin{array}{cccc} x_{i,1} & x_{i,2} & \cdots & x_{i,1024} \end{array}\right]$$

Each column in the training matrix X represents a feature for N molecules or samples, where N is equal to 102,516 molecules.

(12) $$F_{i}=\left[\begin{array}{c} x_{1,i}\\ x_{2,i}\\ x_{3,i}\\ \vdots \\ x_{102516,i} \end{array}\right]$$

Three different structures of the DBN method were used in this study for reweighting molecular features, as described in Section 3.1 and 4.2. The outputs from the DBN methods involved were converted into new matrices of similar size to those of datasets (102,516*1024), although the new matrices represented the newly reconstructed feature weights for the molecules in all input data sets. We used Y to represent the new matrix for the new feature weights of all the molecules:

(13) $$Y=\left[\begin{array}{ccc} y_{1,1} & \cdots & y_{1,M}\\ \vdots & \ddots & \vdots \\ y_{N,1} & \cdots & y_{N.M} \end{array}\right]$$

where $y_{i,j}$ represents the $j^{th}$ feature weight for the $i^{th}$ sample or molecule. The reconstructed features error were calculated by subtracting the final weight of the new feature weights from the original feature weight as E=‖X−Y‖. We used E to presents the reconstructed feature error training matrix, where each value in this matrix, $e_{i,j}$, represents the $j^{th}$ reconstructed feature error for the $i^{th}$ molecule. The main purpose of this dimension reduction is to determine which features have lower error rate and which features have high error rate values. It is very important to select features with lower error values for molecular similarity searching.

(14) $$E=\left[\begin{array}{ccc} e_{1,1} & \cdots & e_{1,M}\\ \vdots & \ddots & \vdots \\ e_{N,1} & \cdots & e_{N.M} \end{array}\right]$$

**Figure 6:**
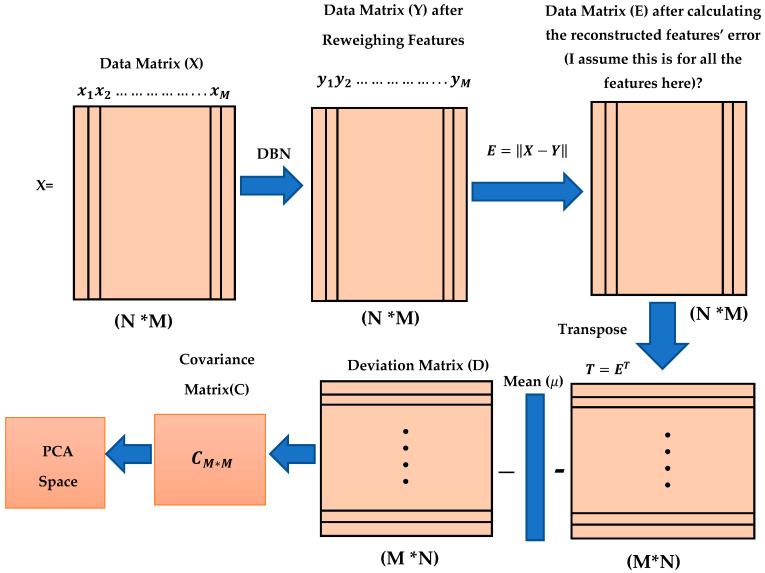
Stages of Proposed methodology.

Prior to implementing PCA, the reconstructed feature error matrix (E) is transformed as follows: $T=E^{T}$ to have new dimensions (M* N) (1024*102,516), where each vector represents one reconstructed feature error for all the molecules in the dataset. Implementing the PCA depends on calculating the covariance matrix (C). Before calculating the covariance matrix (C), we need to calculate the deviation matrix (D) as follows:$\;D_{M*N}=e_{i,j}-\mu_{i}$*,* where $\mu_{i}$ is the mean value of the $i^{th}$ sample and is defined as $\mu_{i}=\sum_{j=1}^{M}e_{j,i}.\;$ The covariance matrix is then calculated as follows: $C_{M*M}=DD^{T}$. Figure 6 summarizes all the proposed methodological steps used in this study, starting from training the DBN to calculate the reconstructed feature’s weight, then calculating the feature’s reconstruction error, after which the deviation matrix and covariance matrix are calculated and the PCA is applied.

**Figure 7:**
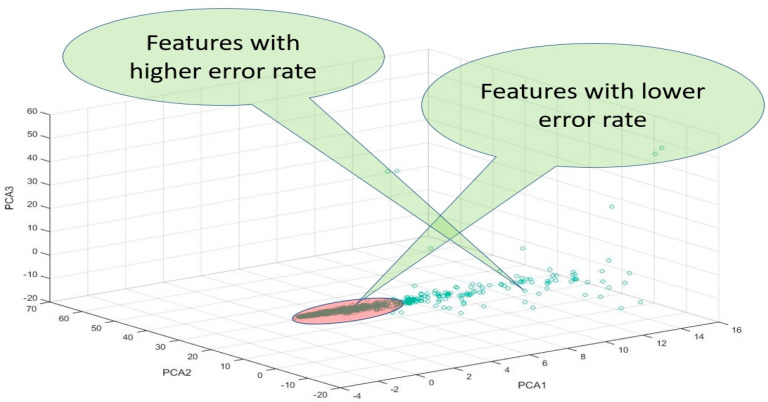
Principal component analysis (PCA) based reconstruction features error.

The PCA space represents the direction of the maximum variance of the given data. This space consists of *k* principal components (PCs) and we used *k* = 3 to obtain PCA1, PCA2, and PCA3 and used the 3D coordinates to draw the features based on the three values, as shown in Figure 7. Only the three values for PCA1, PCA2, and PCA3 of all features were used to depict 3D coordinates, using whichever features were proximate to the original points (0, 0, 0) and featured lower error rates.

Following this, the distances between all features and original points were calculated through D = $\sqrt{{\left(x_{i}-x_{j}\right)}^{2}+{\left(y_{i}-y_{j}\right)}^{2}+{\left(z_{i}-z_{j}\right)}^{2}}$, with the distance to the original points (0, 0, 0), such that $x_{j}=y_{j}=z_{j}=0$; this can be expressed as $D=\sqrt{{\left(x_{i}\right)}^{2}+{\left(y_{i}\right)}^{2}+{\left(z_{i}\right)}^{2}}$.

**Figure 8:**
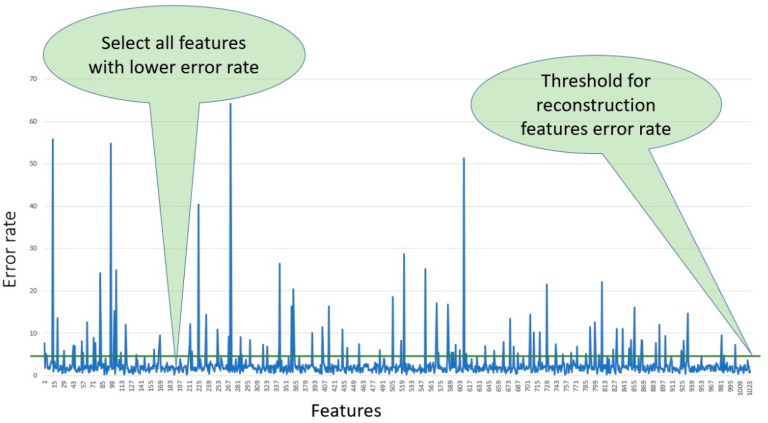
Reconstruction features’ error rates.

In this study, we selected 300 features from each fingerprint, using those features which had lower error rates after filtering all the features according to the percentage of reconstruction feature error and applying the threshold. Figure 8 shows all the features selected based on the error rate and using a threshold value equal to three to select only the features with lowest error rates.

## 4. Experimental Design

To evaluate the performance of the proposed method, we conducted a series of experiments to fulfill the aims of this research, which are (1) What is the performance of the proposed DBN for similarity searching in virtual screening? (2) What is the performance when using stacked DBNs with multi-descriptor features reweighting and selection? (3) How does the proposed SDBN improve the performance of the similarity searches?

MDL Drug Data Report (MDDR) datasets were used to validate the effectiveness of the SDBN method with multi-descriptors, using reweighting of molecular features and feature selection for molecular similarity searching.

### 4.1. Dataset

MDDR collection of datasets [84] remains among the most common databases used in chemo-informatics [85,86]. It comprises 102,516 chemical compounds which include several hundred diverse activities, some of which relate to therapeutic applications, including antihypertensives, while others relate to particular enzymes, including renin inhibitors. The database molecules were converted with Pipeline Pilot ECFC 4 and folded within 1024 bits in size and with corresponding connecting fingerprints extended [81]. All screening experiments used three datasets from the common MDDR database, which are denoted as MDDR-DS1, MDDR-DS2, and MDDR-DS3. Of the eleven activity classes found in MDDR-DS1, some are involved in activities that are homogeneous in structure, whereas others are involved in activities that are heterogeneous in structure (structurally diverse). There are ten are homogeneous activity classes included in the MDDR-DS2 dataset, while the MDDR-DS3 dataset comprises ten heterogeneous activity classes, as shown in Table 1, Table 2 and Table 3.

### 4.2. Evaluation Measures

One of the important measures using a quantitative approach is the Significance Test which is used to measure the performance of the similarity approach. The Kendall W test of concordance is used [87] in this study. Kendall’s W can be translated as the coefficient of concordance, which also known as the measure of agreement among raters. It is assumed that, for each case available, it is either a judge or rater and each variable is either an item or person being judged. The sum of ranks is calculated for each variable. The range of Kendall’s W is between 0 (no agreement) and 1 (complete agreement). Suppose that object *i* (search similarity method) is given the rank $r_{i,j}$ by judge number *j* (activity class), where there are in total *n* objects and *m* judges. Then, the total rank given to the object *i* is

(15) $$R_{i}=\overset{m}{\underset{j=1}{\sum }}r_{i,j}$$

and the mean value of these total rankings is

(16) $$\bar{R}\;\;=\frac{1}{2}m\left(n+1\right)$$

The sum of squared deviations, S, is defined as

(17) $$S=\overset{n}{\underset{i=1}{\sum }}{\left(R_{i}-\bar{R}\right)}^{2}$$

and then Kendall’s W is defined as

(18) $$W=\frac{12\;S}{m^{2}\left(n^{3}-n\right)}$$

The Kendall W test shows whether a set of judges can make comparable judgments about the ranking of a set of objects. In the experiments conducted as part of this paper, the activity classes of each of the data sets were considered as the judges, and the recall rates of the various search models were considered as the objects. The results gave the value of the Kendall coefficient and associated significance levels, which indicates whether the value of the coefficient could have occurred by chance. If the value was significant, (for which cut-off values of both 1% and 5% were set), then it was possible to give an overall ranking to the object. The similarity methods based on the reweighted fragment were also compared to standard methods such as BIN [51], TAN [88], ASMTP [86], and SQB [85]. However, any evaluation of the performance of a specific case depends on the queries, the methods, and the datasets, so all comparisons between methods in this paper were conducted using the same queries and datasets.

### 4.3. Comparison Methods

This section presents various existing methods that serve as the basis for performance evaluation for the proposed model. These include:

- SQB: This is a molecular similarity method that utilizes a quantum mechanics approach. The method specifically relies on the complex pure Hilbert space of molecules for improving the model’s performance.
- ASMTP: This is a similarity measure based on ligand-based virtual screening. The method was designed to utilize a textual database, p, for processing chemical structure databases.
- TAN [88]: This method is widely used in both binary and distance similarity coefficients. Generally, there are two formulae for binary and continuous data, one of which is known as the main molecular similarity method.
- BIN [51]: This serves as an alternative form of calculation used for finding the similarity of molecular fingerprints in ligand-based virtual screening.

## 5. Results and Discussion

Experimental simulations of virtual screenings using MDDR datasets demonstrated that this proposed technique allows various means of improving the efficiency of ligand-based virtual screenings, particularly for more diverse datasets. The MDDR benchmark datasets (MDDR-DS1, MDDR-DS2, and MDDR-DS3) are three different types of datasets chosen from the MDDR database. The MDDR-DS1 includes eleven activity classes, some actives of which are structurally homogeneous while others are structurally heterogeneous. The MDDR-DS2 dataset includes ten homogeneous activity classes, while the MDDR-DS3 data set includes ten heterogeneous activity classes.

From MDDR-DS1, MDDR-DS2, and MDDR-DS3, ten active molecules were randomly selected from each activity class, which are called as reference structures. The similarities between each reference structure and all the molecules in each database were calculated. The results of this similarity were then ranked in decreasing order and only 1% and 5% were selected for each reference structure. The results obtained for each reference structure were investigated to see how many active molecules belonged to the same activity group, referred to as true positive values of the retrieval results. These values were calculated for the ten reference structures, and the average of these values, known as the recall value for the activity class was calculated for the 1% and 5% cutoffs. This procedure was repeated for all datasets. Tables 4, 6, and 8 show the dataset activity classes in the first column, while the other columns show the average of recall values for all activity classes at the cut-off 1%. Tables 5, 7, and 9 show the dataset activity classes in the first column, while the other columns show the average of recall values for all activity classes at the cut-off of 5%. The end of each column shows the overall average recall results of all classes. The best average recall for each class is highlighted. At the bottom of each column, there is a row of shaded cells that corresponds to the total number of shaded cells for all the similarity methods that achieved best results.

Some molecular representations and descriptors are complementary to the others; hence, their combination can yield good results. This indicated that use of different descriptors could yield differing similarity searching results since they incorporate various molecular representations. Based on these reasons, the SDBN model has shown an improvement for the molecular similarity searching based on reweighing and combining different molecular features. The key idea of using SDBN in this work is to learn a rich representation using unsupervised learning to provide a better similarity metric for ligand-based virtual screening. The reconstruction weights for all molecules’ features were obtained, along with PCA to reduce the dimensionality of these molecular features based on selecting the features that have a low error reconstruction error rate and removing the features’ outliers. SDBN was implemented with the important features selected from all the descriptors that were combined to improve the molecular similarity search process.

The results obtained by SDBN are shown in Tables 4–9. The proposed SDBN method is compared with four different benchmark methods that have been used recently for similarity searching, which are BIN, TAN, ASMTP, and SQB.

Table 4 and Table 5 show the results of applying the SDBN proposed method to MDDR-DS1 and the results were then compared with different benchmark methods (BIN, SQB, ASMTP, and TAN). The SDBN was trained with many different architectures and the best results were obtained by using DBN with five RBMs (2000, 1800, 1300, 800, and 300) with 70 epochs, batch size of 128 and learning rate of 0.05 as mentioned in the experimental design section. The results show that SDBN performed better with MDDR-DS1 than all the benchmark similarity methods (BIN, TAN, ASMTP, and SQB) with gains over these methods of 1.7, 2.62, 4.55, and 4.77 percent respectively for overall average results with top 1% of recall results and gains of 3.13, 3.06, 4.72, and 6.06 percent, respectively, for the overall average results with top 5% of recall results. The proposed DSBN method achieved good results when applied to MDDR-DS1, and it outperformed the other methods (BIN, TAN, ASMTP, and SQB). SDBN achieved promising results in eight out of 11 classes with a cut-off of 1% and seven out of 11 with a cut-off of 5%.

The MDDR-DS2 dataset includes ten homogeneous activity classes. The molecules in this dataset are more alike and have low diversity. Table 6 and Table 7 show the results of the recall values of SDBN, which were then compared with different benchmark methods (BIN, TAN, ASMTP, and SQB). Table 6 shows the top 1% retrieval results, where the proposed SDBN method performed better than all the other benchmark similarity methods (BIN, TAN, ASMTP, and SQB) with gains over these methods of 1.06, 1.54, 7.29, and 19.67 percent, respectively, for overall average results. In the overall average results of MDDR-DS2 with the top of 1% of retrieval results, SDBN achieved good results and outperformed the other methods (BIN, TAN, ASMTP, and SQB). The SDBN method achieved good results on the MDDR-DS2 in 5 out of 10 classes with a cutoff of 1%. For the top 5% of recall results, the ASMTP and SQB benchmark methods performed better than SDBN, with gains of 2.68% for ASMTP and 1.93% for SQB.

Nevertheless, SDBN with the top 5% of recall results still showed a good performance in this case and performed better compared with BIN with gains of 2.68% for overall average results and 15.86 % with TAN.

The MDDR-DS3 dataset includes ten heterogeneous activity classes. The molecules in this dataset are highly diverse. The best SDNB results were obtained with the MDDR-DS3 dataset compared with the other two datasets. Table 8 and Table 9 present the results of the SDBN proposed method with MDDR-DS3 compared with those of different benchmarks methods (BIN, SQB, and TAN). The results show that SDBN performed better with MDDR-DS3 than all the other benchmark similarity methods (BIN, SQB, and TAN) with gains of 4.95%, 5.88%, and 6.63% respectively for the overall average results with the top 1% of recall results and gains over the other methods of 6.09%, 6.47%, and 6.04%, respectively, for the overall average results with top 5% of recall results. In the overall average results for MDDR-DS3, SDBN achieved good results and outperformed other methods (BIN, SQB, and TAN). SDBN achieved good results in 9 out of 10 classes with a cut-off of 1% and 10 out of 11 with a cut-off of 5%.

The performance of the similarity methods of the MDDR datasets was ranked by applying Kendall’s W test of concordance. Here, the judge ranking (raters) of the similarity methods (ranked objects) is considered based on the recall values for all activity classes (11 classes for MDDR-DS1, 10 classes each for MDDR-DS2 and MDDR-DS3). The Kendall coefficient (W) and the significance level (p value) are the outputs of this test, where the p value is considered as significant if *p* < 0.05; only then is it possible to give an overall ranking to the similarity methods.

In Table 10 and Table 11, for all used datasets, the results of Kendall W test, and it can be seen that the values associated with probability (p) are less than 0.05. This indicates that for all cases, the results for the SDBN method are significant with cut-off of 1%. As the results show, based on overall ranking of techniques, the SDBN with MDDR-DS1 and MDDR-DS3 at both cut-off of 1% and 5% is superior to BIN, SQB, ASMSC, and TAN. For MDDR-DS2, the BIN method had a higher ranking that the other methods with cut-off of 1% while with cut-off of 5%. The ASMSC provided the best ranking among the methods.

## 6. Conclusions

This study has emphasized the usefulness of deep learning methods for exploring ways to enhance similarity searches in virtual screenings. In addition, the use of deep belief networks associated with the concept of data fusion has been investigated in this study. The aim of this research was to ensure that reliable reconstruction weights for all molecular features were obtainable in several molecular descriptors to reweight molecular features and selected only the important features, i.e., those have more weight with lower error rates and to remove the feature outliers. The feature outliers are those with large reconstruction errors, which can be identified by analyzing the distribution of the reconstruction errors. The experimental results showed that the SDBN with multi-descriptors enhanced the effectiveness of ligand-based virtual screening in chemical databases, establishing that the SDBN can be implemented successfully to enhance the performance of similarity searches. The experiments were conducted based on the MDDR benchmark dataset and revealed the ligand-based virtual screening of chemical databases, to be more effective than the other methods considered. Generally, the screening and evaluation results indicated that the proposed model provides an improvement over other similarity procedures, such as TAN, the SQB method, BIN, and ASMTP. The evaluation of the results achieved from conducting the screening showed that the performance obtained by the proposed measure was improved and, particularly, that the performance of SDBN with the structurally heterogeneous data sets (MDDR -DS1 and MDDR -DS3) achieved superior results compared with the other methods which have been used in previous studies to enhance molecular similarity searching.

## Figures and Tables

**Table 1:** The MDDR-DS1 structure activity classes.

Activity Class	Active Molecules	Activity Index
Renin inhibitors	1130	31,420
HIV protease inhibitors	750	71,523
Thrombin inhibitors	803	37,110
Angiotensin II AT1 antagonists	943	31,432
Substance P antagonists	1246	42,731
5HT3 antagonist	752	06,233
5HT reuptake inhibitors	359	06,245
D2 antagonists	395	07,701
5HT1A agonists	827	06,235
Protein kinase C inhibitors	453	78,374
Cyclooxygenase inhibitors	636	78,331

**Table 2:** The MDDR-DS2 structure activity classes.

Activity Class	Active Molecules	Activity Index
Adenosine (A1) agonists	207	07,707
Adenosine (A2) agonists	156	07,708
Renin inhibitors	1130	31,420
CCK agonists	111	42,710
Monocyclic β-lactams	1346	64,100
Cephalosporins	113	64,200
Carbacephems	1051	64,220
Carbapenems	126	64,500
Tribactams	388	64,350
Vitamin D analogous	455	75,755

**Table 3:** The MDDR-DS3 structure activity classes.

Activity Class	Active Molecules	Activity Index
Muscarinic (M1) agonists	900	09,249
NMDA receptor antagonists	1400	12,455
Nitric oxide synthase inhibitors	505	12,464
Dopamine β-hydroxylase inhibitors	106	31,281
Aldose reductase inhibitors	957	43,210
Reverse transcriptase inhibitors	700	71,522
Aromatase inhibitors	636	75,721
Cyclooxygenase inhibitors	636	78,331
Phospholipase A2 inhibitors	617	78,348
Lipoxygenase inhibitors	2111	78,351

**Table 4:** Retrieval results of top 1% for MDDR-DS1 dataset.

Activity Index	SDBN	BIN	SQB	ASMSC	TAN
31,420	74.21	74.08	73.73	73.84	69.69
71,523	27.97	28.26	26.84	15.03	25.94
37,110	26.03	26.05	24.73	20.82	9.63
31,432	39.79	39.23	36.66	37.14	35.82
42,731	23.06	21.68	21.17	19.53	17.77
06,233	19.29	14.06	12.49	10.35	13.87
06,245	6.27	6.31	6.03	5.5	6.51
07,701	14.05	11.45	11.35	7.99	8.63
06,235	12.87	10.84	10.15	9.94	9.71
78,374	17.47	14.25	13.08	13.9	13.69
78,331	9.93	6.03	5.92	6.89	7.17
**Mean**	**24.63**	22.93	22.01	20.08	19.86
**Shaded cells**	**8**	3	0	0	0

**Table 5:** Retrieval results of top 5% for MDDR-DS1 dataset.

Activity Index	SDBN	BIN	SQB	ASMSC	TAN
31,420	89.03	87.61	87.75	86	83.49
71,523	65.17	52.72	60.16	51.33	48.92
37,110	41.25	48.2	39.81	23.87	21.01
31,432	79.87	77.57	82	76.63	74.29
42,731	31.92	26.63	28.77	32.9	29.68
06,233	29.31	23.49	20.96	26.2	27.68
06,245	21.06	14.86	15.39	15.5	16.54
07,701	28.43	27.79	26.9	23.9	24.09
06,235	27.82	23.78	22.47	23.6	20.06
78,374	19.09	20.2	20.95	22.26	20.51
78,331	16.21	11.8	10.31	15	16.2
**Mean**	**40.83**	37.70	37.77	36.11	34.77
**Shaded cells**	**7**	1	1	2	0

**Table 6:** Retrieval results of top 1% for MDDR-DS2 dataset.

Activity Index	SDBN	BIN	SQB	ASMSC	TAN
07,707	83.19	72.18	72.09	67.86	61.84
07,708	94.82	96	95.68	97.87	47.03
31,420	79.27	79.82	78.56	73.51	65.1
42,710	74.81	76.27	76.82	81.17	81.27
64,100	93.65	88.43	87.8	86.62	80.31
64,200	71.16	70.18	70.18	69.11	53.84
64,220	68.71	68.32	67.58	66.26	38.64
64,500	75.62	81.2	79.2	46.24	30.56
64,350	85.21	81.89	81.68	68.01	80.18
75,755	96.52	98.06	98.02	93.48	87.56
**Mean**	**82.30**	81.24	80.76	75.01	62.63
**Shaded cells**	**5**	3	0	1	1

**Table 7:** Retrieval results of top 5% for MDDR-DS2 dataset.

Activity Index	SDBN	BIN	SQB	ASMSC	TAN
07,707	73.9	74.81	74.22	76.17	70.39
07,708	98.22	99.61	100	99.99	56.58
31,420	95.64	65.46	95.24	95.75	88.19
42,710	90.12	92.55	93	96.73	88.09
64,100	99.05	99.22	98.94	98.27	93.75
64,200	93.76	99.2	98.93	96.16	77.68
64,220	96.01	91.32	90.9	94.13	52.19
64,500	91.51	94.96	92.72	90.6	44.8
64,350	86.94	91.47	93.75	98.6	91.71
75,755	91.6	98.35	98.75	97.27	94.82
**Mean**	91.68	90.70	93.61	**94.36 **	75.82
**Shaded cells**	1	3	2	**4**	0

**Table 8:** Retrieval results of top 1% for MDDR-DS3 dataset.

Activity Index	SDBN	BIN	SQB	TAN
09,249	19.47	15.33	10.99	12.12
12,455	13.29	9.37	7.03	6.57
12,464	12.91	8.45	6.92	8.17
31,281	23.62	18.29	18.67	16.95
43,210	14.23	7.34	6.83	6.27
71,522	11.92	4.08	6.57	3.75
75,721	29.08	20.41	20.38	17.32
78,331	11.93	7.51	6.16	6.31
78,348	9.17	9.79	8.99	10.15
78,351	18.13	13.68	12.5	9.84
**Mean**	**16.38**	11.43	10.50	9.75
**Shaded cells**	**9**	1	0	0

**Table 9:** Retrieval results of top 5% for MDDR-DS3 dataset.

Activity Index	SDBN	BIN	SQB	TAN
09,249	31.61	25.72	17.8	24.17
12,455	16.29	14.65	11.42	10.29
12,464	20.9	16.55	16.79	15.22
31,281	36.13	28.29	29.05	29.62
43,210	22.09	14.41	14.12	16.07
71,522	14.68	8.44	13.82	12.37
75,721	41.07	30.02	30.61	25.21
78,331	17.13	12.03	11.97	15.01
78,348	26.93	20.76	21.14	24.67
78,351	17.87	12.94	13.3	11.71
**Mean**	**24.47**	18.38	18.00	18.43
**Shaded cells**	**10**	0	0	0

**Table 10:** The results of Kendall W test for DS1 and DS2 datasets.

Dataset	Recall Type	W	P	SDBN	BIN	SQB	ASMSC	TAN
MDDR-DS1	1%	0.321	0.021	4.727	4.091	2.273	2.000	1.909
	5%	0.613	0.0036	4.364	2.727	2.818	2.818	2.273
MDDR-DS2	1%	0.521	0.0013	3.8	4.1	3.3	2.5	1.6
	5%	0.715	0.00016	2.7	3.5	3.7	3.8	1.3

**Table 11:** The results of Kendall W test for DS1 and DS2 dataset.

Dataset	Recall Type	W	P	SDBN	BIN	SQB	TAN
MDDR-DS3	1%	0.496	0.006	3.8	2.9	1.7	1.6
	5%	0.318	0.004	4	2	2	2

## Data Availability

The MDL Drug Data Report (MDDR) dataset is owned by www.accelrys.com. A license is required to access the data.

## Sample Availability

Samples of the compounds are available from the authors.
